# Dietary flavonoids of Spanish youth: intakes, sources, and association with the Mediterranean diet

**DOI:** 10.7717/peerj.3304

**Published:** 2017-05-17

**Authors:** Rowaedh Ahmed Bawaked, Helmut Schröder, Lourdes Ribas-Barba, Gabriela Cárdenas, Luis Peña-Quintana, Carmen Pérez-Rodrigo, Montserrat Fíto, Lluis Serra-Majem

**Affiliations:** 1 Cardiovascular Risk and Nutrition Research Group (CARIN), Hospital del Mar Medical Research Institute (IMIM), Barcelona, Spain; 2 Department of Experimental and Health Sciences, University of Pompeu Fabra, Barcelona, Spain; 3 CIBER Epidemiology and Public Health (CIBERESP), Instituto de Salud Carlos III, Madrid, Spain; 4 Fundación para la Investigación Nutricional (Nutrition Research Foundation), Barcelona, Spain; 5 CIBER Physiopathology of Obesity and Nutrition (CIBEROBN), Instituto de Salud Carlos III, Madrid, Spain; 6 Reseach Institute of Biomedical and Health Sciences, University of Las Palmas de Gran Canaria, Gran Canaria, Spain; 7 FIDEC Foundation, University of the Basque Country (UPV/EHU) Bilbao, Bilbao, Spain; 8 Department of Nutrition, Food Sciences and Gastronomy, University of Barcelona, Barcelona, Spain

**Keywords:** Flavonoids, Mediterranean diet, Children, Adolescents, KIDMED index, Flavonoids intake, Plant-based diets, Flavan-3-ols

## Abstract

**Background:**

Plant-based diets have been linked to high diet quality and reduced risk of cardiovascular diseases. The health impact of plant-based diets might be partially explained by the concomitant intake of flavonoids. Estimation of flavonoids intake in adults has been important for the development of dietary recommendations and interventions for the prevention of weight gain and its consequences. However, estimation of flavonoids intake in children and adolescents is limited.

**Methods:**

Average daily intake and sources of flavonoids were estimated for a representative national sample of 3,534 children and young people in Spain, aged 2–24 years. The data was collected between 1998 and 2000 by 24-h recalls. The Phenol-Explorer database and the USDA database on flavonoids content were used. Adherence to the Mediterranean diet was measured by the KIDMED index.

**Results:**

The mean and median intakes of total flavonoids were 70.7 and 48.1 mg/day, respectively. The most abundant flavonoid class was flavan-3-ols (35.7%), with fruit being the top food source of flavonoids intake (42.8%). Total flavonoids intake was positively associated with the KIDMED index (*p* < 0.001).

**Conclusion:**

The results of this study provide primary information about flavonoids intake and main food sources in Spanish children, adolescents and young adults. Participants with high daily mean intake of flavonoids have higher adherence to the Mediterranean diet.

## Introduction

Flavonoids, a large group of polyphenolic compounds in plants, are the most abundant polyphenols in the human diet. Over 5,000 hydroxylated polyphenolic compounds have been isolated and identified (Shashank & Abhay, 2013). Dietary flavonoids are classified into six subclasses according to their chemical structure: anthocyanidins, flavonols, flavanones, flavones, isoflavones and flavan-3-ols.

Observational studies have repeatedly suggested a beneficial health effect of flavonoids intake, although they are inconclusive (Mursu et al., 2008; Vogiatzoglou et al., 2015). Flavonoids intake has been associated with reducing the risk of major chronic diseases, such as cardiovascular diseases (Knekt et al., 2002; Kim, Vance & Chun, 2016b), some types of cancers (Hui et al., 2013; Geybels et al., 2013), Alzheimer and diabetes (Zamora-Ros et al., 2013a). Furthermore, increased intake of most flavonoid subclasses has shown inverse relationship with weight gain in men and women aged 27–65 years (Bertoia et al., 2016), with incidence of hypertension in middle-aged women (Lajous et al., 2016), and with risk of depression in older women (Chang et al., 2016).

The effect of a plant-based diet on the risk of chronic diseases and mortality may partly be due to flavonoid compounds and other plant metabolites (Watzl, 2008). Flavonoids have been found to decrease inflammation, scavenge free radicals (Shashank & Abhay, 2013), and improve vascular endothelial function (Hooper et al., 2008). Although descriptive studies in adult populations have estimated dietary flavonoids intake, data on flavonoids intake in children and adolescents is scarce despite growing evidence of the preventive effects of dietary flavonoids.

High intake of plant-based foods such as fruits and vegetables is an essential characteristic of high diet quality and linked to favorable health outcomes (Hu, 2003; Crowe et al., 2011). The Mediterranean diet is a traditional plant-based dietary pattern, well known for its high vegetables and fruit intake and overall healthy eating pattern (Sebastian et al., 2015b; Sebastian et al., 2015a). The health impact of plant-based diets might be partially explained by the concomitant intake of flavonoids, but evidence on the association between flavonoids intake and diet quality is scarce in adult populations (Sebastian et al., 2015b) and essentially nonexistent in younger people. Similarly, the association between Mediterranean dietary pattern and flavonoids intake has not been studied in children.

Knowledge of population-based flavonoids intake pattern is important for the development of dietary recommendations. Additionally, the identification of dietary patterns related to increased flavonoids consumption is relevant because of the potential impact of these bioactive compounds in disease prevention. Therefore, this study aimed to estimate the intake of flavonoids; describe major sources of flavonoids; and evaluate the association between flavonoids intake and diet quality i.e., measured by the adherence to Mediterranean diet; among Spanish children, adolescence and young adults.

## Methods

### Study design

The enKid Study, described in detail elsewhere (Serra-Majem et al., 2003), was a cross-sectional survey of the nutritional status and food habits of 3,534 Spanish children and young adults, carried out between 1998 and 2000. Participants were selected by multistage random sampling procedures based on a population register. The objective of the EnKid study was two-fold: (1) to establish the prevalence of micronutrient deficiencies in the population aged 2–24 years; and (2) to analyze the association of these micronutrients with group membership by sex and age. The sample size of 3,850 participants was calculated according to the estimated prevalence of most micronutrients, with 95% confidence interval and accuracy of ±2.5% of the average value of the micronutrient, and a statistical power of 80% to detect significant differences between two groups (=10% of the mean of the micronutrients, setting the alpha error at *p* = 0.05). Taking into account an anticipated 70% participation rate, we oversized the study sample size to 5,500 participants (anticipated prevalence of non-participation: *n* = 1,650 (30%)). Recruitment achieves a sample of 3,534 individuals. Parental written consent was obtained on behalf of each participant younger than 18 years. The ethics committee of the Spanish Society of Community Nutrition approved the study protocol.

### Dietary and lifestyle data collection

Dietary data was collected during in-home interviews carried out by 43 trained dietitians and nutritionists using household measures to estimate portion sizes. Dietary intake was assessed by 24-hour recall. The same field staff entered survey data into software specifically designed for the study. The 24-hour recall did not include herbs and spices.

The nutrient database software used for the study consisted of a Spanish database (Mataix Verdú, Aranceta Batrina & Serra-Majem, 1995), completed with information from French and British (Holland et al., 1991) food composition tables.

Participants responding to an interviewer-administered questionnaire reported data on maternal education, which was recorded in five categories: (i) no education (never went to school), (ii) primary education not completed, (iii) completed primary education, (iv) secondary education, and (v) university.

### Flavonoid food composition database

The Phenol-Explorer database, a comprehensive database on polyphenol content in foods, was the main source for flavonoid, isoflavone, and proanthocyanin content used in estimating dietary flavonoid intake in the enKid study. This database has been extracted from more than 1,300 scientific publications and includes 35,000 content values for 500 different polyphenols in more than 400 foods (Rothwell et al., 2013). In addition, US Department of Agriculture (USDA) databases were used to determine the flavonoids content of foods not available from Phenol-Explorer data (Bhagwat, Haytowitz & Holden, 2011). Flavonoid intakes from food were measured by multiplying the consumption frequency of each food by its flavonoid content. Flavonoid intake from foods with several varieties was averaged. For example, flavonoid content of “apples” was averaged for the two types of apples listed in Phenol Explorer, cider apples and whole raw dessert apples.We measured the flavonoid intake of the six subclasses: flavonols (quercetin, kaempferol, myricetin, and isorhamnetin), flavones (luteolin and apigenin), flavanones (eriodictyol, hesperetin, and naringenin), flavan-3-ols (catechin, gallocatechin, epicatechin, epigallocatechin, epicatechin-3-gallate, epigallocatechin-3-gallate, theaflavin, theaflavin 3-gallate, theaflavin 3′-gallate, theaflavin 3,3′ digallate, and thearubigins), anthocyanidins (cyanidin, delphinidin, malvidin, pelargonindin, petunidin, and peonidin), and isoflavones (daidzein, genistein, and glycitein). Total flavonoids were calculated as the sum of these subclasses.

### KIDMED index

Adherence to the Mediterranean diet was estimated by the KIDMED index, derived from a 16-item questionnaire administered separately from the 24-hour recalls as part of the enKid survey (Serra-Majem et al., 2004). The KIDMED index was created specifically to estimate adherence to the Mediterranean diet in children and young adults, based on the principles that sustain the Mediterranean dietary pattern and those that undermine it. Four items denoting lower adherence were assigned a value of −1 (Goes more than once a week to a fast-food restaurant; skips breakfast; has commercially baked goods or pastries for breakfast; takes sweets and candy several times every day) and the 12 items related to higher adherence were scored +1 (Takes a fruit or fruit juice every day; has a second fruit every day; regularly has fresh or cooked vegetables once a day; has fresh or cooked vegetables more than once a day; consumes fish regularly; likes pulses and eats them more than once a week; consumes pasta or rice almost every day (five or more times per week); has cereals or grains (bread, etc.) for breakfast; consumes nuts regularly (at least 2–3 times per week); uses olive oil at home; has a dairy product for breakfast (yoghurt, milk, etc.); takes two yoghurts and/or some cheese (40 g) daily). Scores range from −4 to 12, with higher scores indicating greater adherence to the Mediterranean diet. A low, intermediate, and high adherence to the Mediterranean diet was defined as scoring below 4, between 4 and 7, and more than 7 points for the KIDMED index, respectively.

### Statistical analysis

General linear modeling procedures were used to compare baseline participant characteristics by quintiles of total flavonoids intake. Polynomial contrast was used to determine overall *p* for linear trend for continuous variables with normal distribution, and Kruskal-Wallis test to determine overall *p* for non-normal distributions. *P* for linear trend for categorical variables was obtained by Mantel-Haenszel linear-by-linear association chi-square test.

To determine the association between flavonoids and the KIDMED index, we fitted general linear models adjusted for sex, age, maternal education level, energy intake, region, and community size. Polynomial contrast was used to determine overall *p* for linear trend. Associations were considered significant if *P* < 0.05. The SPSS for Windows version 18 (SPSS, Inc., Chicago, IL, United States) was used for all statistical analysis.

## Results

Participants with high Flavonoid intake were more likely to be female, were older, and had higher maternal education level. Daily energy intake and adherence to the Mediterranean diet increased across the total flavonoids intake (Table 1).

**Table 1:** Main characteristics of study participants according to quartiles of total flavonoid intake.

	Q1	Q2	Q3	Q4	*P*^a^
	*N* = 843	*N* = 782	*N* = 819	*N* = 818	
Male *n* (%)	392 (46.5)	347 (44.4)	387 (47.3)	383 (46.8)	<0.001
Age (years)	15.51 (6.4)	14.65 (6.4)	14.88 (6.36)	16.35 (6.17)	<0.001
Maternal education level *n* (%)^b^	558 (66.8)	538 (69.5)	573 (70.6)	563 (69.8)	<0.001
Energy intake (Kcal/day)	1915 (741)	2042 (719)	2122 (777)	2217 (894)	<0.001
Kidmed score (%)	6.65 (2.19)	7.13 (2.07)	7.39 (1.94)	7.62 (2.06)	<0.001
Community size *n* (%)^c^					0.001
<10,000	200 (23.7)	167 (21.4)	159 (19.4)	193 (23.6)	
10,000–50,000	231 (27.4)	206 (26.3)	199 (24.3)	209 (25.6)	
50,000–350,000	225 (26.7)	214 (27.4)	234 (28.6)	201 (24.6)	
>350,000	187 (22.2)	195 (24.9)	227 (27.7)	215 (26.3)	
Region *n*, (%)^d^					<0.001
Central	220 (26.1)	176 (22.5)	181 (22.1)	211 (25.8)	
Northeast	218 (25.9)	183 (23.4)	194 (23.7)	202 (24.7)	
North	172 (20.4)	180 (23.0)	200 (24.4)	172 (21.0)	
South and Canary Islands	129 (15.3)	156 (19.9)	145 (17.7)	133 (16.3)	
East	104 (12.3)	87 (11.1)	99 (12.1)	100 (12.2)	

**Notes.**

a *p* values were obtained by ANOVA, Kruskal Wallis, and Pearson chi-square for normal continuous, non-normal continuous, and categorical variables, respectively; mean and standard deviation for continuous variables age, energy intake; proportions within quintiles for categorical variables (male sex, community size, and region).

b Maternal education above primary school.

c Percentage expressed as proportion within community size.

d Percentage expressed as proportion within region.

The mean and median intake of total flavanols for children and young adults aged 2–24 years in Spain was 70.7 and 48.1 mg/day, respectively. The mean intake was twice the median intake of total flavonoids, indicating skewed distributions to low values. The mean intake of flavan-3-ols was 25.2 mg/day, representing the main contributor to total flavonoids intake by (35.7%), followed by flavanones at 19.7 mg/day (27.8%), flavonols at 15.6 mg/day (22.1%), and anthocyanins at 7.7 mg/day (10.9%). The lowest contributions were from flavones, 2.2 mg/day (3.2%), and isoflavones, 0.1 mg/day (1.2%) (Table 2).

**Table 2:** Flavonoid intake of Spanish youth.^a^

Age	Flavonoids intake (mg/day)					
	Mean	Median	SD	25th	50th	75th
Total flavonoids						
All	70.7	48.1	84.1	19.3	48.0	93.1
2–5	54.7	41.4	56.5	17.7	41.4	76.8
6–11	62.3	48.5	54.4	23.7	48.5	86.4
10–13	65.1	44.4	75.6	18.8	44.4	89.3
18–24	82.4	51.8	102.8	18.9	51.8	105.7
Flavonols						
All	15.6	5.9	30.6	1.8	5.9	17.2
2–5	10.3	4.4	18.4	1.4	4.4	11.0
6–11	11.7	4.3	20.3	1.9	4.3	12.6
12–17	14.1	4.9	28.0	1.5	4.9	15.9
18–24	19.6	8.2	37.4	2.3	8.2	22.8
Flavones						
All	2.2	0.3	9.1	0.0	0.3	1.1
2–5	1.6	0.3	3.8	0.0	0.3	1.6
6–11	1.6	0.3	5.8	0.0	0.4	1.2
12–17	1.8	0.2	7.1	0.0	0.2	0.9
18–24	2.9	0.3	11.9	0.0	0.3	1.1
Flavanones						
All	19.7	0.1	34.1	0	0.1	28.1
2–5	16.7	8.7	24.4	0	8.7	27.7
6–11	18.2	0.1	28.9	0	0.1	28.1
12–17	17.2	0.1	32.0	0	0.07	25.3
18–24	22.8	0.3	39.7	0	0.3	28.2
Flavan-3-ols						
All	25.2	14.1	47.1	4.7	14.1	28.1
2–5	21.0	13.4	34,6	6.3	13.4	24.6
6–11	23.8	19.0	24.6	9.3	19.0	30.1
12–17	24.4	15.6	44.7	6.1	15.6	27.9
18–24	27.5	11.2	57.6	2.2	11.2	27.8
Anthocyanins						
All	7.7	0.3	27.1	0	0.3	4.2
2–5	4.9	0.5	12.4	0	0.5	6.4
6–11	6.5	0.4	22.1	0	0.4	4.7
12–17	7.3	0.0	28.6	0	0.0	3.5
18–24	9.3	0.5	30.4	0	0.5	4.8
Isoflavones						
All	0.1	0	1.4	0	0	0
2–5	0.1	0	1.8	0	0	0
6–11	0.1	0	2.1	0	0	0
10–13	0.0	0	0.1	0	0	0
18–24	0.1	0	1.3	0	0	0

**Notes.**

a Intake is expressed as mean and median with standard deviation (SD) and quartile distribution.

In stratified analysis adjusted for age, maternal education level, energy intake, region and community size, flavonoids intake was greater among girls, and increased with age. The mean total flavonoids intake for girls was 72.1 mg/day, and for boys was 69.1 mg/day. The mean intake of total flavonoids was 81.5 mg/day in the age group “18–24 years”, 62.9 mg/day in “12–17 years”, 63.5 mg/day in “6–11 years”, and 61.8 mg/day for the age group 2–5 years old.

The major dietary sources of total flavonoids intake were fruits (including fruit juices), vegetables, and chocolate products (Table 3). Fruits were the major contributors, accounting for 42.8% of daily total flavonoids intake. Vegetables, particularly spinach, onions, artichokes and lettuce contributed 22% of the daily total; the highest flavonoids intake from vegetables was in the older age group (18–24 years). Cocoa powder and chocolate contributed 23.5% of total flavonoid intake (Table 3), with the highest daily intake among children aged 6–11 years.

**Table 3:** Contribution of different food groups to total flavonoids intakes.

Food groups	Mean flavonoid intake (mg/d)	SD	Proportion of intake (%)	Major food sources of total flavonoid intake
Fruits and fruit juices	38.2	56.8	42.8	Oranges
				Natural fruit Juice
				Apples
				Commercial fruit juice
Vegetables	11.1	29.1	22.0	Spinach
				Onions
				Artichokes
				Green beans/ Lettuce
Chocolate	10.9	31.7	23.5	Cocoa powder
				Instant cocoa powder
				Dark chocolate
				Milk chocolate
Alcoholic beverages	0.66	19.4	0.31	Red wine
(2–17) years old				Sangria
Alcoholic beverages	11.2	58.6	6.0	
(18–24) years old				
Legumes	3.40	14.3	4.6	White beans
				Red beans
				Lentils
Non-alcoholic beverages	1.64	20.5	1.7	Tea
				Apple cider
Nuts	0.08	0.86	0.4	Pistachios
				Almonds
Oil and fat	0.08	0.15	1.4	Virgin olive oil
				Olive oil

After adjusting for sex, age, maternal education, energy intake, region, and community size, the mean of total flavonoids intake was positively associated with the KIDMED index score. The same was true for all flavonoid compounds except for flavones (Table 4).

**Table 4:** Association between flavonoid intake and adherence to the Mediterranean diet measured by the KIDMED index.^a^

KIDMED score	Poor < 3		Medium 4–7		High > 10		*P*^b^
	Mean	95% CI	Mean	95% CI	Mean	95% CI	
Total flavonoids	50.76	35.9–65.54	63.6	59.9–67.39	80.8	76.2–85.4	<0.001
Flavonols	12.24	6.88–17.6	13.6	12.3–15.0	18.1	16.3–19.8	<0.001
Flavones	1.64	0.10–3.19	2.07	1.62–2.53	2.43	1.99–2.86	0.3
Flavanones	8.30	3.50–13.1	17.6	16.0–19.3	23.1	21.3–24.9	<0.001
Flavan-3-ols	21.5	13.8–29.2	23.8	21.6–5.32	27.5	24.9–29.9	0.05
Anthocyanins	7.02	2.42–11.6	6.36	5.32–7.41	9.54	7.90–11.1	0.01
Isoflavones	0.03	0.00–0.05	0.04	0.01–0.07	0.15	0.05–0.25	0.08

**Notes.**

a General linear models adjusted for sex, age, region, community size, maternal education and energy intake.

b *p* values were obtained by ANOVA test.

## Discussion

This study explored the flavonoids intake in the Spanish youth population of the ENKID study. The estimated mean total flavonoids intake was 70.7 mg/d, with flavan-3-ols as the main subclass and fruits and fruit juice as the major dietary source. Energy-adjusted flavonoids intake was slightly higher in girls than boys, and increased with age. Higher adherence to the Mediterranean diet was correlated with higher flavonoids intake.

Data on flavonoids intake in children are limited. In comparison with previous studies, the mean intake in this population; i.e., 70.7 mg/day for ages 2–24 years, was lower than the intake reported for the US NHANSE 2007–2010 study, i.e., 108.5 mg/day for ages 2–19 years (Sebastian et al., 2016). Based on age groups, total flavonoid intakes for the NHANSE study were 59.5 mg/d for ages 2–5 years, 79.8 mg/d for ages 6–11 years, and 153.6 mg/d for ages 12–17 years (Sebastian et al., 2015a).

Comparing flavonoids intake between different studies could be influenced by several factors; for example, by the dietary assessment method, study sample, and the flavonoid database. An Australian study estimated the intake of flavonoids in children, adolescences and young adult used a single 24HR dietary assessment, similar to our study (Johannot & Somerset, 2006). The study reported a mean flavonoids intake of 24.0–181.0 mg/d in 2–24 years old Australians (Johannot & Somerset, 2006). Comparing to our findings in small age groups 2–5 and 6–11 year-old intakes were around 62 mg/d, the intake in the Australian study similar age groups is considerably lower.

In the NHANSE study, flavonoids intake was higher in boys than girls (Sebastian et al., 2016). In our study, however, flavonoids intake was higher in boys than girls only before adjusting for energy intake. A study by Yngve et al. (2005) found that children from Spain and Iceland had the lowest fruit and vegetables consumption, and that, among 11-year-olds, boys consumed less vegetables and fruits than girls in five out of nine European countries—but in Spain, boys consumed slightly more than girls.

Similar to our study, flavan-3-ols were the most abundant class of dietary flavonoids in children and adults in the US (Kim, Vance & Chun, 2016a). In the Australian study, flavan-3-ols accounted for 75% of flavonoids intake for the population aged 2 years and older, and up to 92% in adults aged 19 years and older. Australians’ high level of tea consumption contributed 67% of flavonoids intake (Johannot & Somerset, 2006). This finding explains the noticeable difference in flavonoids intake between the Australian population aged 19–24 years and the same age group in our study. After excluding tea flavonoids, the Australian population aged 19–24 years decreased in flavonoids intake from more than 200 mg/day to less than 50 mg/day (Johannot & Somerset, 2006).

Tea was also the major source of flavonoids intake in a US adult population (Kim, Vance & Chun, 2016a). Tea contributed 155.9 mg/d of daily total flavonoids intake in the US adults, compared to 2.3 mg/day in the young adults of our study. One explanation for this extreme difference is that the EnKid dietary data were collected between 1998 and 2000, a period when tea was consumed in low quantities in the Spanish population (Consortium et al., 2012). In the Spanish EPIC study, tea intake contributed 2.2% (6.8 mg/d) of total flavonoids intake (Zamora-Ros et al., 2010).

In Mediterranean countries, fruits and wine are the principal sources for flavonoids intake in adults (Zamora-Ros et al., 2013b). The findings of the present study confirm the role of fruits as the main dietary contributors to flavonoids intake in children, adolescents, and young adults. Our results are in line with the Spanish EPIC study finding that fruits were the main source of flavonoids in younger Mediterranean populations (Zamora-Ros et al., 2010).

Comparisons of flavonoid intakes between different populations and time periods have certain limitations, depending on the degree of consistency in the flavonoid data base used, the dietary assessment method selected, and the timing of the dietary assessments. Our study used a recently developed flavonoid database (Bhagwat, Haytowitz & Holden, 2011; Rothwell et al., 2013), which was not available when most of the cited studies were carry out. According to the most recent report of food consumption in Spain, intake of fruit and vegetables has decreased (Ministerio de Agricultura y Pesca, 2015). Therefore, it is reasonable to assume that current flavonoid intakes of Spanish youth are lower than those reported in the EnKid study (Johannot & Somerset, 2006). Similarly, the percentage of the Australian adults not meeting the recommended intake of vegetables and fruits has increased (Canberra: National Health and Medical Council, 2013). Our study used the KIDMED index as a measure of diet quality to test our hypothesis that flavonoids intake is related to higher diet quality in youth. All classes of flavonoids except flavones were positively associated with KIDMED index. Our results support a previous study which found an association between consuming the recommended servings of fruits and vegetables and significantly higher phytonutrient intake, including flavonoids intake, compared to participants who did not meet the recommended consumption levels (Murphy et al., 2012). Other studies have found that diet quality, measured by the healthy-eating index (HEI), was positively associated with flavonoids intake and nearly all healthy-eating index components increased across quartiles of flavonoids intake (Sebastian et al., 2015b; Sebastian et al., 2015a).

A limitation of the present study is its cross-sectional design, which precludes drawing causal relationships. Furthermore, 24-hour recalls—and particularly a single day’s recall—have inherent limitations in the individual assessment of dietary intake due to daily variations in food intake. However, the sample size of 3,534 participants was large enough to characterize group intakes. Major strengths of our study are the nationwide population-based sample and interviewer-guided completion of validated questionnaires.

In conclusion, this study provides the first basic data for estimated flavonoids intake and their sources in Spanish youth. Fruits were the main source of dietary flavonoids. Flavan-3-ol was the major contributor for total flavonoids intake. Flavonoids intake was positively associated with good diet quality, indicated by higher adherence to the Mediterranean diet.

##  Supplemental Information

10.7717/peerj.3304/supp-1 Supplemental Information 1 Raw data exported from SPSS for the flavonoids data analysis

## Additional Information and Declarations

### Data Availability

The following information was supplied regarding data availability: The raw data has been supplied as a Supplementary File.

## References

[ref-1] Bertoia ML, Rimm EB, Mukamal KJ, Hu FB, Willett WC, Cassidy A (2016). Dietary flavonoid intake and weight maintenance: three prospective cohorts of 124 086 US men and women followed for up to 24 years. BMJ.

[ref-2] Bhagwat S, Haytowitz DB, Holden JM (2011). https://www.ars.usda.gov/northeast-area/beltsville-md/beltsville-human-nutrition-research-center/nutrient-data-laboratory/docs/usda-database-for-the-flavonoid-content-of-selected-foods-release-31-december-2013/.

[ref-3] Canberra: National Health and Medical Council, R. National Health and Medical Research Council (2013). Australian dietary guidelines.

[ref-4] Chang SC, Cassidy A, Willett WC, Rimm EB, O’Reilly EJ, Okereke OI (2016). Dietary flavonoid intake and risk of incident depression in midlife and older women. American Journal of Clinical Nutrition.

[ref-5] Consortium TI, Wild S, Roglic G, Green A, Sicree R, King H, Abdullah A, Peeters A, Courten M de, Stoelwinder J, Manikandan R, Sundaram R, Thiagarajan R, Sivakumar M, Meiyalagan V, Lenzen S, Kao Y, Chang H, Lee M, Chen C, Jing Y, Han G, Hu Y, Bi Y, Li L, Sartorelli D, Fagherazzi G, Balkau B, Touillaud M, Boutron-Ruault M, Oba S, Nagata C, Nakamura K, Fujii K, Kawachi T, Takatsuka N, Dieren S Van, Uiterwaal C, Schouw Y van der, der A van, Boer J, Boggs D, Rosenberg L, Ruiz-Narvaez E, Palmer J, Huxley R, Lee C, Barzi F, Timmermeister L, Czernichow S, Riboli E, Hunt K, Slimani N, Ferrari P, Norat T, InterAct CT, Wareham N, Jakes R, Rennie K, Schuit J, Mitchell J, Onland-Moret N, der A van, Schouw Y van der, Buschers W, Elias S, Higgins J, Thompson S, Fukino Y, Ikeda A, Maruyama K, Aoki N, Okubo T, Hosoda K, Wang M, Liao M, Chuang C, Iha M, Huyen V, Phan D, Thang P, Hoa N, Ostenson C, Nagao T, Meguro S, Hase T, Otsuka K, Komikado M, Ryu O, Lee J, Lee K, Kim H, Seo J, Stendell-Hollis N, Thomson C, Thompson P, Bea J, Cussler E, Carlsen M, Halvorsen B, Holte K, Bohn S, Dragland S, Lakenbrink C, Lapczynski S, Maiwald B, Engelhardt U, Kyle J, Morrice P, McNeill G, Duthie G, Arts M, Haenen G, Wilms L, Beetstra S, Heijnen C, Hollman P, Hof K Van Het, Tijburg L, Katan M, Leenen R, Roodenburg A, Tijburg L, Wiseman S, Hof K Van Het, Wiseman S, Yang C, Tijburg L, Langley-Evans S, Serafini M, Ghiselli A, Ferro-Luzzi A, Grove K, Lambert J (2012). Tea consumption and incidence of type 2 diabetes in europe: the EPIC-InterAct Case-Cohort study. PLOS ONE.

[ref-6] Crowe FL, Roddam AW, Key TJ, Appleby PN, Overvad K, Jakobsen MU, Tjønnel A, Hansen L, Boeing H, Weikert C, Linseisen J, Kaaks R, Trichopoulou A, Misirli G, Lagiou P, Sacerdote C, Pala V, Palli D, Tumino R, Panico S, Bueno-De-Mesquita HB, Boer J, Van Gils CH, Beulens JWJ, Barricarte A, Rodríguez L, Larrañaga N, Sánchez M-J, Tormo M-J, Buckl G, Lund E, Hedblad B, Melander O, Jansson J-H, Wennberg P, Wareham NJ, Slimani N, Romieu I, Jenab M, Danesh J, Gallo V, Norat T, Riboli E (2011). Fruit and vegetable intake and mortality from ischaemic heart disease: results from the European Prospective Investigation into cancer and nutrition (EPIC)-Heart study. European Heart Journal.

[ref-7] Geybels MS, Verhage BAJ, Arts ICW, Van Schooten FJ, Alexandra Goldbohm R, Van Den Brandt PA (2013). Dietary flavonoid intake, black tea consumption, and risk of overall and advanced stage prostate cancer. American Journal of Epidemiology.

[ref-8] Holland B, Widdowson EM, Unwin ID, Buss DH (1991). Vegetables, herbs and spices: fifth supplement to McCance and widdowson’s the composition of foods.

[ref-9] Hooper L, Kroon PA, Rimm EB, Cohn JS, Harvey I, Cornu KA Le, Ryder JJ, Hall WL, Cassidy A (2008). Flavonoids, flavonoid-rich foods, and cardiovascular risk: a meta-analysis of randomized controlled trials. The American Journal of Clinical Nutrition.

[ref-10] Hu FB (2003). Plant-based foods and prevention of cardiovascular disease: an overview. American Journal of Clinical Nutrition.

[ref-11] Hui C, Qi X, Qianyong Z, Xiaoli P, Jundong Z, Mantian M (2013). Flavonoids, flavonoid subclasses and breast cancer risk: a meta-analysis of epidemiologic studies. PLOS ONE.

[ref-12] Johannot L, Somerset SM (2006). Age-related variations in flavonoid intake and sources in the Australian population. Public Health Nutrition.

[ref-13] Kim K, Vance TM, Chun OK (2016a). Estimated intake and major food sources of flavonoids among US adults: changes between 1999–2002 and 2007–2010 in NHANES. European Journal of Nutrition.

[ref-14] Kim K, Vance TM, Chun OK (2016b). Greater flavonoid intake is associated with improved CVD risk factors in US adults. British Journal of Nutrition.

[ref-15] Knekt P, Kumpulainen J, Järvinen R, Rissanen H, Heliövaara M, Reunanen A, Hakulinen T, Aromaa A (2002). Flavonoid intake and risk of chronic diseases. The American Journal of Clinical Nutrition.

[ref-16] Lajous M, Rossignol E, Fagherazzi G, Perquier F, Scalbert A, Clavel-Chapelon F, Boutron-Ruault MC (2016). Flavonoid intake and incident hypertension in women. American Journal of Clinical Nutrition.

[ref-17] Mataix Verdú J, Aranceta Batrina J, Serra-Majem L (1995). Tabla de composición de alimentos españoles.

[ref-18] Ministerio de Agricultura y Pesca AyMA (2015). Informe del consumo de alimentación en España.

[ref-19] Murphy MM, Barraj LM, Herman D, Bi X, Cheatham R, Randolph RK (2012). Phytonutrient intake by adults in the United States in relation to fruit and vegetable consumption. Journal of the Academy of Nutrition and Dietetics.

[ref-20] Mursu J, Nurmi T, Tuomainen T-P, Salonen JT, Pukkala E, Voutilainen S (2008). Intake of flavonoids and risk of cancer in finnish men: the kuopio ischaemic heart disease risk factor study. International Journal of Cancer.

[ref-21] Rothwell JA, Perez-Jimenez J, Neveu V, Medina-Remón A, M’Hiri N, García-Lobato P, Manach C, Knox C, Eisner R, Wishart DS, Scalbert A (2013). Phenol-Explorer 3.0: a major update of the Phenol-Explorer database to incorporate data on the effects of food processing on polyphenol content. Database.

[ref-22] Sebastian RS, Wilkinson Enns C, Goldman JD, Martin CL, Steinfeldt LC, Murayi T, Moshfegh AJ (2015a). A new database facilitates characterization of flavonoid intake, sources, and positive associations with diet quality among US adults. Journal of Nutrition.

[ref-23] Sebastian R, Wilkinson Enns C, Goldman J, Moshfegh A (2015b). Flavonoid intakes are predictive of dietary quality and most components of the healthy eating index 2010. FASEB Journal.

[ref-24] Sebastian RS, Wilkinson Enns C, Goldman JD, Steinfeldt LC, Martin CL MA (2016). Flavonoid values for USDA survey foods and beverages 2007–2010.

[ref-25] Serra-Majem L, Ribas-Barba L, Aranceta Bartrina J, Pérez-Rodrigo C, Saavedra Santana P, Peña Quintana L (2003). Obesidad infantil y juvenil en España. Resultados del Estudio enKid (1998–2000). Medicina Clínica.

[ref-26] Serra-Majem L, Ribas L, Ngo J, Ortega RM, Garcia A, Perez-Rodrigo C, Aranceta J (2004). Food, youth and the Mediterranean diet in Spain. Development of KIDMED, mediterranean diet quality index in children and adolescents. Public Health Nutrition.

[ref-27] Shashank K, Abhay K (2013). Review article chemistry and biological activities of flavonoids: an overview. The Scientific World Journal.

[ref-28] Vogiatzoglou A, Mulligan AA, Bhaniani A, Lentjes MAH, McTaggart A, Luben RN, Heiss C, Kelm M, Merx MW, Spencer JPE, Schroeter H, Khaw KT, Kuhnle GGC (2015). Associations between flavan-3-ol intake and CVD risk in the Norfolk cohort of the European prospective investigation into cancer (EPIC-Norfolk). Free Radical Biology and Medicine.

[ref-29] Watzl B (2008). Anti-inflammatory effects of plant-based foods and of their constituents. International Journal for Vitamin and Nutrition Research.

[ref-30] Yngve A, Wolf A, Poortvliet E, Elmadfa I, Brug J, Ehrenblad B, Franchini B, Haraldsdóttir J, Krølner R, Maes L, Pérez-Rodrigo C, Sjöström M, Thórsdóttir I, Klepp K-I (2005). Fruit and vegetable intake in a sample of 11-year-old children in 9 European countries: the pro children cross-sectional survey. Annals of Nutrition and Metabolism.

[ref-31] Zamora-Ros R, Andres-Lacueva C, Lamuela-Raventós RM, Berenguer T, Jakszyn P, Barricarte A, Ardanaz E, Amiano P, Dorronsoro M, Larrañaga N, Martínez C, Sánchez MJ, Navarro C, Chirlaque MD, Tormo MJ, Quirós JR, González CA (2010). Estimation of dietary sources and flavonoid intake in a Spanish adult population (EPIC-Spain). Journal of the American Dietetic Association.

[ref-32] Zamora-Ros R, Forouhi NG, Sharp SJ, González CA, Buijsse B, Guevara M, Schouw YT, Amiano P, Boeing H, Bredsdorff L, Clavel-Chapelon F, Fagherazzi G, Feskens EJ, Franks PW, Grioni S, Katzke V, Key TJ, Khaw KT, Kühn T, Masala G, Mattiello A, Molina-Montes E, Nilsson PM, Overvad K, Perquier F, Quirós JR, Romieu I, Sacerdote C, Scalbert A, Schulze M, Slimani N, Spijkerman AMW, Tjonnel A, Tormo MJ, Tumino R, Van Der ADL, Langenberg C, Riboli E, Wareham NJ (2013a). The association between dietary flavonoid and lignan intakes and incident type 2 diabetes in european populations: the EPIC-InterAct study. Diabetes Care.

[ref-33] Zamora-Ros R, Knaze V, Luján-Barroso L, Romieu I, Scalbert A, Slimani N, Hjartåker A, Engeset D, Skeie G, Overvad K, Bredsdorff L, Tjønnel A, Halkjær J, Key TJ, Khaw K-T, Mulligan AA, Winkvist A, Johansson I, Bueno-de Mesquita HB, Peeters PHM, Wallström P, Ericson U, Pala V, de Magistris MS, Polidoro S, Tumino R, Trichopoulou A, Dilis V, Katsoulis M, Huerta JM, Martínez V, Sánchez M-J, Ardanaz E, Amiano P, Teucher B, Grote V, Bendinelli B, Boeing H, Förster J, Touillaud M, Perquier F, Fagherazzi G, Gallo V, Riboli E, González CA (2013b). Differences in dietary intakes, food sources and determinants of total flavonoids between Mediterranean and non-Mediterranean countries participating in the European Prospective Investigation into Cancer and Nutrition (EPIC) study. The British Journal of Nutrition.

